# Supplementary material for: Vitamin D Modified DSS-Induced Colitis in Mice via STING Signaling Pathway
Source: Biology (Basel). 2025 Jun 18;14(6):715. doi: 10.3390/biology14060715 (PMC12190092; doi:10.3390/biology14060715)
Supplement: Supplementary file 1 [file biology-14-00715-s001.zip › Supplementary Materials S1: 16s-report of gut microbiota/Groups/diff_analysis/metagenomeRGenus/figure/barplot/alllog.pdf]

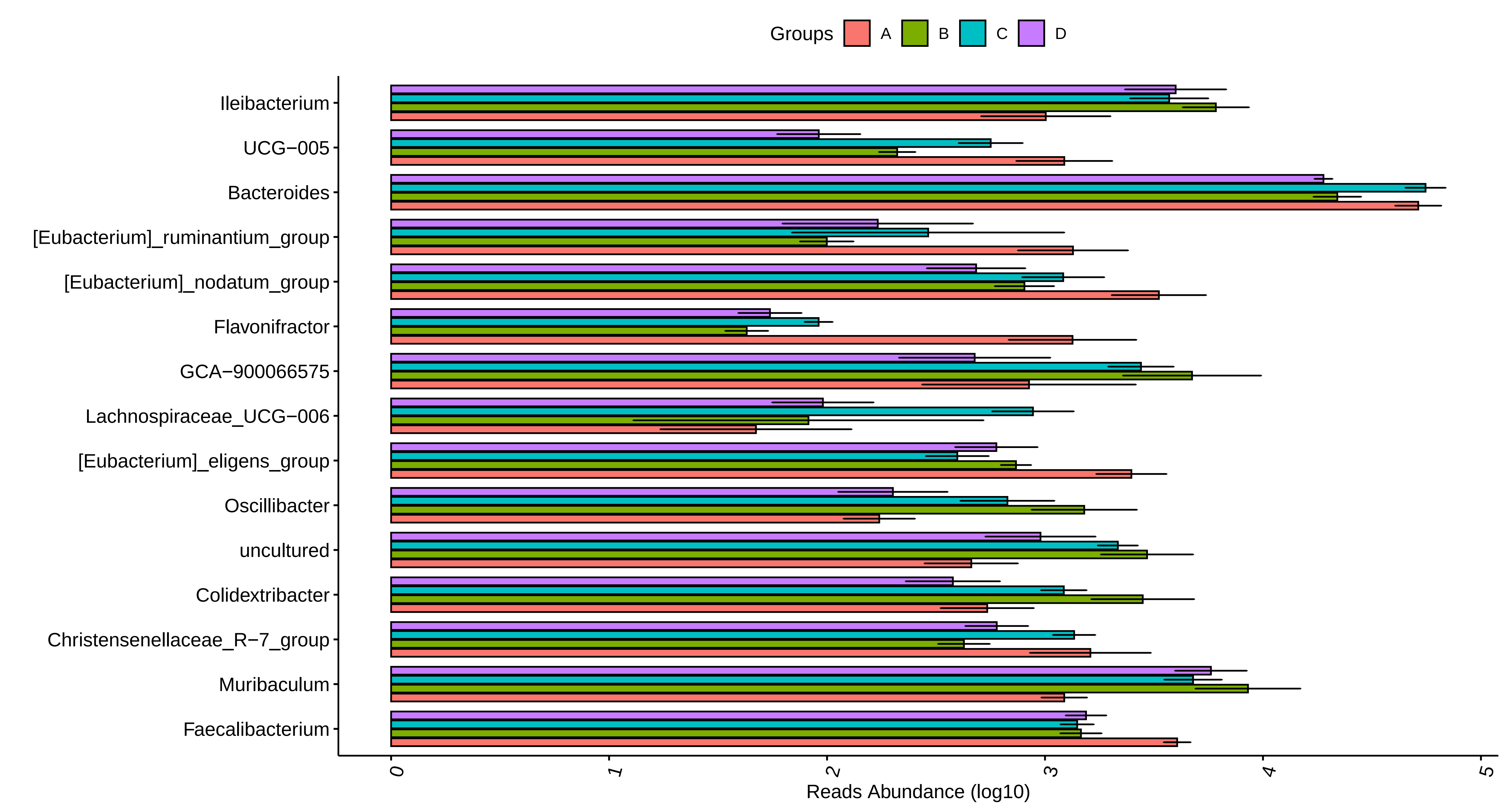
Groups
A
B
C
D
Ileibacterium
UCG−005
Bacteroides
[Eubacterium]_ruminantium_group
[Eubacterium]_nodatum_group
Flavonifractor
GCA−900066575
Lachnospiraceae_UCG−006
[Eubacterium]_eligens_group
Oscillibacter
uncultured
Colidextribacter
Christensenellaceae_R−7_group
Muribaculum
Faecalibacterium
0
1
2
3
4
5
Reads Abundance (log10)
